# Manual of Chemical Tests, and of Chemical Decomposition

**Published:** 1839-04

**Authors:** 


Art. VIII.—*Handbuch der Reagentien-und Zerlegungslehre, &c.* Von Hofrath Dr. Du Menil.—*Lemgo*, 1836-7.

*Manual of Chemical Tests, and of Chemical Decomposition.* By Dr. Du Menil.—*Lemgo*, 1836-7. Two vols. pp. 729.

This work which has been only lately completed, is novel in its plan; and we have seldom seen one presenting in the same space so great an amount of useful chemical knowledge. The author has entirely reversed the ordinary method of treating the subject, a circumstance, however, of little importance, when the plan of the work is fully understood. His main object has been to bring into as small a space as possible, all the tests or reagents capable of acting upon any simple substance, or any of the compounds formed by it. The two volumes are divided into fifty-three chapters, a chapter being devoted to each simple body. In the first volume we have the metalloids and the alkaline and earthy metals; in the second we have the true metals. The difference between the author's work and a common treatise on chemistry may be judged of from this brief statement. We will take for illustration the chapter on Mercury. In a chemical treatise all the chemical properties of this metal and its salts would be described. Here, however, it is presumed that the reader already possesses to a certain extent a knowledge of these. Dr. Du Menil prefixes to this chapter, the symbols of the metal, its most striking properties, and a condensed table with the centesimal composition of its oxides, chlorides, and sulphurets. Then follow the reagents under distinct sections, to the number of thirty-four: the difference, if any, in the action of these upon the salts of the two degrees of oxidation, as well as the comparative minuteness or delicacy of each of the most important; so that the point at which the test fails to detect the metal is numerically stated. Frequent experiment has convinced us that if the author have in some few instances exaggerated the effects of these, his statements are in the main correct and may be relied on. The rules for quantitative analysis are given under the appropriate sections; and the chapter is terminated by a minute account of the action of heat upon the metal and all of its compounds. It is the same with every other metal. The immense mass of information contained in these two volumes, would of course be lost unless there were easy means of reference. We admire the simplicity with which this end has been most effectually attained. At the conclusion of each chapter there is an alphabetical index, by which each reagent may be immediately found through the figures attached to the sections. Besides this, in the second volume we have a most copious index occupying seventy-eight pages of close type, in which every reagent in the two volumes is again marked down alphabetically; and references given to its separate action upon all the metals and their salts. Further, if the reader wish to know how to separate any two bodies from each other, he has only to refer to a second index following the first, expressly adapted for quantitative analysis. We shall conclude our notice of this work by saying that it is a monument of the industry and learning of the author. The preparation of it must have cost him some years of labour: but we trust he will meet his reward in the well merited reputation which it must acquire for him, both in this country and on the continent.

